# Parameters for Successful Parental RNAi as An Insect Pest Management Tool in Western Corn Rootworm, *Diabrotica virgifera virgifera*

**DOI:** 10.3390/genes8010007

**Published:** 2016-12-24

**Authors:** Ana M. Vélez, Elane Fishilevich, Natalie Matz, Nicholas P. Storer, Kenneth E. Narva, Blair D. Siegfried

**Affiliations:** 1 Department of Entomology, University of Nebraska, 103 Entomology Hall, Lincoln, NE 68583, USA; nataliematz88@gmail.com; 2 Dow AgroSciences, 9330 Zionsville Road, Indianapolis, IN 46268, USA; EFishilevich@dow.com (E.F.); nstorer@dow.com (N.P.S.); KNarva@dow.com (K.E.N.); 3 Entomology and Nematology Department, University of Florida, Charles Steinmetz Hall, PO Box 110620, Gainesville, FL 32611, USA; bsiegfried1@ufl.edu

**Keywords:** rootworm, *Diabrotica*, RNAi, parental RNAi, insect resistance, *brahma*, *hunchback*, chromatin-remodeling ATPase, transgenic crops

## Abstract

Parental RNAi (pRNAi) is an RNA interference response where the gene knockdown phenotype is observed in the progeny of the treated organism. pRNAi has been demonstrated in female western corn rootworms (WCR) via diet applications and has been described as a potential approach for rootworm pest management. However, it is not clear if plant-expressed pRNAi can provide effective control of next generation WCR larvae in the field. In this study, we evaluated parameters required to generate a successful pRNAi response in WCR for the genes *brahma* and *hunchback*. The parameters tested included a concentration response, duration of the dsRNA exposure, timing of the dsRNA exposure with respect to the mating status in WCR females, and the effects of pRNAi on males. Results indicate that all of the above parameters affect the strength of pRNAi phenotype in females. Results are interpreted in terms of how this technology will perform in the field and the potential role for pRNAi in pest and resistance management strategies. More broadly, the described approaches enable examination of the dynamics of RNAi response in insects beyond pRNAi and crop pests.

## 1. Introduction

Corn rootworms (CRW), *Diabrotica* species, are the most important pests of maize in the United States Corn Belt, with western corn rootworm (WCR), *Diabrotica virgifera virgifera*, being economically the most impactful [1]. The CRW larval stages cause economic damage by feeding on maize roots; root feeding by *D. v. virgifera* and the northern corn rootworm, *Diabrotica barberi* results in yield losses and costs of control that have been estimated to exceed $1 billion annually [2,3]. However, it is thought that this figure is underestimated today given the ongoing insecticide resistance problems, increased chemical costs, and technology fees associated with transgenic maize varieties [4]. Within the current management strategies, plant-expressed insecticidal proteins from *Bacillus thuringiensis* (Bt) have vastly changed the landscape of CRW control. Bt proteins expressed in maize provide significant root protection against CRW species, protecting yields, and are also believed to ameliorate the impacts of environmental stress conditions, such as drought, that are exacerbated by rootworm pressure [5,6].

RNA interference (RNAi) is recognized as a new potential management tool for this insect through feeding on plants producing long RNA hairpins (hpRNA) that suppress specific target genes in CRW [7]. WCR larvae and adults are generally susceptible to RNAi via ingestion of artificially produced double-stranded RNA (dsRNA) and hpRNA expressed in maize plants [7,8,9]. RNAi is likely to represent a next generation of biotechnological innovations for rootworm management [7,8,10,11]. Transgenic maize targeting WCR via RNAi will complement existing management practices including chemical insecticides and Bt traits [12,13].

To date, most genes proposed as RNAi targets for WCR cause lethality in the larval stage [7,8]. However, the sensitivity of WCR adults to RNAi was recently leveraged to produce phenotypes in larval progeny, referred to as parental RNAi (pRNAi). pRNAi has been described in coleopteran insects [13,14,15,16] as well as in other insect orders [17,18,19,20,21,22,23,24,25]. pRNAi functions by adult feeding on dsRNA that targets genes that regulate embryonic development resulting in reduced egg hatch rates or complete absence of viable larvae, while adults remain unaffected [16,26]. We recently evaluated the pRNAi effects of chromatin remodeling ATPase genes *brahma*, *mi-2*, *iswi-1*, and *iswi-2*, and the gap gene *hunchback* in WCR [16,26]. pRNAi has the potential to be used as part of integrated pest management (IPM) and insect resistance management (IRM) programs in combination with Bt toxins and related technologies to aid in slowing the emergence of alleles conferring resistance to the Bt toxins [26]. Moreover, by reducing the larval infestation in a maize field, pRNAi unique mode of action could potentially preserve the durability of other products used to manage WCR.

To fully evaluate the utility of pRNAi for pest and resistance management, key biological parameters need to be evaluated, including concentration-response, effects of the duration of the exposure, and effects of the timing of exposure within the adult lifecycle. With respect to the dose and exposure time, Bolognesi et al., 2012 [8] evaluated the effect of RNAi in WCR larvae: exposures of two to 24 h showed a response that was dependent on time of exposure and concentration in a 12 day assay. This concentration and time of exposure relationship illustrates that it is necessary to identify these parameters to achieve a consistent and effective RNAi response. Phenotypic responses to varying concentrations have also been documented in WCR larvae using genes critical for cuticle pigmentation [27]. While the effective concentration of dsRNA may be different depending on the target transcript, the sequence of dsRNA, and the stability of the encoded protein, the requirements for a pRNAi response in WCR progeny may further complicate the relationship between exposure and response. Further, since genes targeted by pRNAi affect embryonic development, it is necessary to determine the stage of female reproductive development at which the pRNAi effect is most successful. Ultimately, these parameters may be correlated to the conditions in the field when females are actively consuming maize tissues. With adults being much more mobile than larvae, establishing exposure requirements is critical for ensuring effective pRNAi concentrations in plants expressing hpRNA.

WCR have a univoltine life cycle; eggs are typically laid from late to July to early September and diapause in the soil [3,28]. Egg hatch varies depending on soil temperature; in the Midwest larvae typically hatch between May and early June [29], and feed underground on maize roots. Adults emerge during the summer and are present in and around maize fields from late June to autumn frost [28,30]. The larvae feed continuously for three to four weeks only on the roots of grasses (Graminae), especially maize [31,32]. Adults are strongly attracted to pollen and reproductive plant parts. They feed mainly on maize [4] as well as on other crops such as cucurbits, alfalfa, and soybeans [33,34,35], and pollen of non-crop flowers including *Ambrosia*, *Helianthus*, and *Amaranthus* [36,37].

WCR males emerge approximately five days before females [38]. However, the male emergence period overlaps with the females′, since approximately five to seven days are required for the males to reach sexual maturity [39], while the females are sexually mature upon emergence [40]. In the field, males often intercept teneral virgin females (within 12–24 h of emergence, with pale and soft bodies) shortly after emergence from the soil [41,42,43]; mating couples are commonly observed at the base of maize plants. WCR females usually mate only once and they do not mate again as long as they are actively laying eggs [43]. Females feed on maize tissues available in the field where they emerged before mating or immediately after mating [4]. Female post-emergence dispersal prior to mating is believed to be minimal (1–5 m) and is dependent of whether there are sufficient numbers of males present at emergence [4,44], whereas dispersal after mating can be significant (<1 m/flight to as long as 24 km/flight) [45,46]. Several days after mating, 15% to 24% of the females engage in “sustained” or migratory flights [45,46]. Later studies showed that approximately 70% of females take flights (“trivial or “sustained”) after mating, most of the flights (85%–90%) are of less than 1 min in duration, with only 0.5% of the female flights lasting longer than 20 min [47]. Campbell and Meinke [36] reported that WCR adults frequently move between a maize-prairie interface primarily after corn pollination, when it becomes less attractive than the adjoining prairie. WCR movement is also affected by changes in crop phenology within and among fields [48]. WCR female movement increases in later maize vegetative stages [49,50] and adults tend to move from early-planted maize to late planted maize [46]; adult movement is also density-dependent [51]. The above studies suggest that movement and feeding behavior could influence adult exposure to pRNAi in asynchronous fields with different traits or pRNAi fields adjacent to prairies. Based on the WCR behaviors described above, interplay between exposure duration and parental effect could also affect the success of refuge-based resistance management strategies that are intended to delay the onset of resistance to the pRNAi and Bt proteins in WCR populations [13]. This highlights the importance of identifying the duration of exposure to dsRNA or hpRNA, necessary to generate a pRNAi response and how adult movement will affect this exposure. WCR female feeding and mating behaviors also suggest that females could be exposed to hpRNA at different times of the reproductive cycle indicating the importance of evaluating the timing of exposure required for a successful pRNAi response.

This study aimed to identify the parameters required for a successful pRNAi for two genes in WCR, the chromatin remodeling gene *brahma* (*brm*), and the gap gene *hunchback* (*hb*). The parameters explored in the current work included: (1) a dsRNA concentration response; (2) duration of the dsRNA exposure; and (3) timing of the dsRNA exposure with respect to the mating status in WCR females. The concentration required to generate a pRNAi response with six exposures over twelve days was 0.2 μg/pellet or higher for both *brm* and *hb*. An exposure of four days for *brm* and eight days of *hb* of 2 µg of dsRNA/food pellet (highest amount used; equivalent to ~1.1 µg/insect/day) were necessary to achieve pRNAi responses in WCR. Further, recent work demonstrates that exposure of WCR females to *brm* homologs or *hb* dsRNA significantly affects larval emergence [16,26], however, the effect of *brm* and *hb* on the fecundity and fertility of adult WCR males has not been determined. In this study, we evaluated the effects of *brm* and *hb* dsRNA on male sperm viability and fecundity. Exposure of WCR males to *brm* and *hb* dsRNA had a subtle effect on sperm counts but no detectable effect on the number of offspring produced. The results obtained in this study further characterize the potential effectiveness of in planta pRNAi expression as a pest management tool for rootworm.

## 2. Materials and Methods

### 2.1. Gene Identification

WCR transcriptome sequencing and gene identification was described previously [16,52]. The amino acid sequences of *brahma* (*brm*) and *hunchback* (*hb*) from *Tribolium* were used as query sequences to search the WCR transcriptome. The GenBank accession numbers for WCR sequences for *brm* and *hb* are KR152260 and KR152261, respectively [16].

### 2.2. cDNA Preparation and dsRNA Synthesis

cDNA preparation and dsRNA synthesis was performed as previously described [16,26]. Briefly, total RNA was isolated from non-diapausing WCR adults (Crop Characteristics Inc., Farmington, MN, USA) using RNeasy Mini Kit (Qiagen, Valencia, CA, USA). Total RNA (1 µg) was used to synthesize first strand cDNA using the Quantitech Reverse Transcription Kit (Qiagen) and DNA was amplified using Takara Taq DNA Polymerase (Clontech Laboratories, Inc. Mountain View, CA, USA). All primers contained a T7 promoter sequence at their 5′ ends to enable T7 transcription (Supplementary Materials Table S1) [16]. *Green Fluorescent Protein* (*GFP*) dsRNA was used as a negative control. *Brm*, *hb,* and *GFP* PCR products were used as templates for in vitro synthesis of dsRNAs using the MEGAscript^TM^ T7 RNAi Kit (Ambion, Life Technologies, Carlsbad, CA, USA) and purified using the RNeasy Mini Kit (Qiagen). The dsRNA products were quantified using a NanoDrop^TM^ 100 spectrophotometer (Thermo Scientific, Franklin, MA, USA) at 260 nm and analyzed by gel electrophoresis to determine purity.

### 2.3. pRNAi Phenotypes in Embryos and Ovaries

WCR embryos from females fed with *hb* dsRNA and ovaries of females fed with diet treated with water, GFP, *brm* and *hb* dsRNA for 12 days before or after mating, were dissected under a Leica Zoom 200 stereomicroscope (Leica, Wetzlar, Germany) and stored in 70% ethanol. Images were captured with an Olympus SZX16 microscope, Olympus SDF PLAPO 2X PFC lens and the Olympus CellSens Dimensions software (Olympus, Tokyo, Japan).

### 2.4. brahma and hunchback Concentration Response

Test insects were purchased from Crop Characteristics (Farmington, MN, USA). In each treatment, ten females and ten males (24–48 h old) were maintained on untreated artificial diet and allowed to mate for four days in 16-well trays (5.1 cm long × 3.8 cm wide × 2.9 high) with vented lids. The artificial diet was adapted from Branson and Jackson [53] to provide the consistency necessary to cut diet plugs that could be treated with dsRNA. Diet was poured into Petri dishes to a depth of approximately 0.5 cm and after solidification the diet plugs (~4 mm in diameter × 2 mm height) were cut from the diet with a #1 (4 mm) cork borer. Trays were held in a growth chamber at 23 ± 1 °C, relative humidity >80%, and 16:8 L:D photoperiod [16]. Four replications of ten females and ten males were completed per treatment.

Four days after mating, males were removed and the remaining females were provided with eleven diet plugs surface-treated with gene specific dsRNA. WCR females were exposed to four concentrations of *brm* or *hb* dsRNA, 2 µg, 0.2 µg, 0.02 µg, and 0.002 µg per diet plug. Water and 2 µg of *GFP* dsRNA served as the controls. Freshly-treated diet was provided every other day, for a total of six exposures over twelve days. On Day 10 of exposure, females were transferred to polystyrene oviposition egg boxes (7.5 cm × 5.5 cm × 5.5 cm) (ShowMan box, Althor Products, Wilton, CT, USA) using the design of Campbell and Meinke [54]. The boxes contained moistened silty clay loam soil, pre-sifted through a 60-mesh sieve and autoclaved [55]. Females were allowed to lay eggs for four days, then were removed and flash frozen for qRT-PCR. Eggs were incubated in soil within the oviposition boxes for ten days at 27 °C, relative humidity >80% and 24 h dark. Eggs were removed from the soil by washing through a 60-mesh sieve. Harvested eggs were held in Petri dishes on moistened filter paper at 28 °C, relative humidity >80%, 24 h dark. The Petri dishes were photographed and total eggs counted using the cell counter function of ImageJ software [56]. The number of larvae hatching from each plate was recorded daily for fifteen days to determine egg viability [16].

### 2.5. Duration of brahma and hunchback dsRNA Exposure

To identify the duration of exposure necessary to generate a pRNAi response, females were exposed to 2 µg of dsRNA/plug one, two, four, or six times. The methodology used for this experiment was similar to that described for the concentration response experiment. Briefly, ten females and ten males (24–48 h old) were maintained on untreated artificial diet and allowed to mate for four days. After mating, males were removed and females were transferred to new trays with eleven dsRNA- or control-treated diet plugs. Freshly treated diet was provided every other day for eleven days but unlike the concentration response experiment, females were exposed one, two, four, or six times to 2 µg of *brm* or *hb* dsRNA per diet plug. Untreated artificial diet was provided for the remaining days. The controls, water and 2 µg of *GFP* dsRNA, were provided six times. After four days in the oviposition boxes, females were flash frozen for qRT-PCR. Eggs were washed, placed in Petri dishes, imaged, and analyzed with ImageJ, as described above. Larval hatching was monitored daily for fifteen days to determine egg viability. Four replicates of ten females and ten males were completed per treatment.

### 2.6. Timing of brahma and hunchback dsRNA Exposure with Respect to Mating Status

Previous experiments evaluated the pRNAi response for *brm* and *hb* in females exposed to dsRNA immediately after mating [16]. To determine if WCR female sensitivity to pRNAi varies with age and mating status, females were exposed to dsRNA six times prior to mating, immediately after mating, and six days after mating. The methodology used for this experiment was similar to that described for the concentration and duration response experiments.

Four replications of ten females and ten males per replication were completed for each type of exposure. The evaluation of the pRNAi effects immediately after mating was used as a reference and was performed using the methods described for the concentration response experiment. Briefly, artificial diet was surface-treated with water or 2 µg of *brm*, *hb* or *GFP* dsRNA six times over eleven days. After oviposition in oviposition boxes, females were flash frozen for qRT-PCR. Eggs were washed, placed in Petri dishes, imaged, and analyzed with ImageJ. Larval hatching was monitored daily for fifteen days to determine egg viability.

To determine the pRNAi effects in females before mating, ten virgin females (24–48 h old) were fed artificial diet treated with water or 2 µg of *brm*, *hb* or *GFP* dsRNA six times over eleven days. On Day 12 females were paired with ten virgin males and provided with untreated diet. Four days after mating, males were removed and females were transferred to trays with untreated diet. Females were transferred to oviposition boxes after six days, allowed to lay eggs for four days then removed and flash frozen for qRT-PCR. Eggs were washed, placed in Petri dishes, imaged, and analyzed with ImageJ. Larval hatching was monitored daily for fifteen days to determine egg viability.

To evaluate the pRNAi effect after mating, ten females and ten males (24–48 h old) were allowed to mate for four days. After mating, males were removed and females were transferred to trays with untreated diet. Females were provided untreated diet every other day for five days. Six days after mating, females were transferred to trays with artificial diet surface-treated with water or 2 µg of *brm*, *hb* or *GFP* dsRNA six times over eleven days. Females were transferred to oviposition boxes the day of the second exposure to dsRNA. One day after the last exposure, females were removed and flash frozen for qRT-PCR. Eggs were washed, placed in Petri dishes, imaged, and analyzed with ImageJ. Larval hatching was monitored daily for fifteen days to determine egg viability.

### 2.7. Effects of brahma and hunchback on Males

The effect of pRNAi in males was evaluated by exposing virgin males to artificial diet treated with dsRNA before mating. Ten virgin males (24–48 h old) were fed eleven pellets of artificial diet treated with water or 2 µg of specific *brm*, *hb* or *GFP* dsRNA. Freshly treated diet was provided every other day for seven days for a total of four exposures. On Day 8, three males per replication per treatment were flash frozen for qRT-PCR and the remaining males were paired with ten virgin females. Males were removed after four days and females were transferred to trays with untreated diet. Six days after mating females were transferred to oviposition boxes and allowed to lay eggs for four days. Eggs were washed, placed in Petri dishes, imaged, and analyzed with ImageJ. Larval hatching was monitored daily for fifteen days to determine egg viability. Three replications per treatment were performed. A second experiment with six exposures to dsRNA and three replications was performed to evaluate the effect on oviposition, egg hatching, and relative gene expression.

Sperm viability was evaluated in live males after four exposures to dsRNA over eight days. One day after the last exposure four males per replication were evaluated. Sperm viability was assessed using the Live/Dead Sperm Viability Kit (Invitrogen, Carlsbad, CA, USA) to discriminate between living and dead sperm [57]. WCR males were anesthetized on ice, testes and seminal vesicles were dissected under a stereomicroscope, placed in 10 μl of buffer (HEPES 10 mM, NaCl 150 mM, BSA 10%, pH 7.4) and crushed with a toothpick. Immediately after dissection, 1 μL of SYBR 14 (0.1 mM in dimethyl sulfoxide (DMSO)) was added and incubated at room temperature for ten minutes, followed by 1 μL of propidium iodine (2.4 mM) and incubated again at room temperature for ten minutes. Ten microliters of the sperm stained solution was transferred to a glass microslide and evaluated using a Nikon Eclipse 90i microscope with a Nikon A1 confocal and NIS-Elements Software (Melville, NY, USA). Samples were visualized at 10× with 488 excitation, a 500–550 nm band pass for live sperm (SYBR 14) and 663–738 nm band pass for dead sperm (propidium iodine) simultaneously. Digital images were recorded for five fields of view per sample. The numbers of live (green) and dead (red) sperm were evaluated using the cell counter function of ImageJ [56].

### 2.8. Quantitative Real-Time PCR (qRT-PCR)

WCR qRT-PCR was performed using SYBR green and the 7500 Fast System Real-Time PCR System (Applied Biosystems, Foster City, CA, USA). Total RNA isolation and cDNA preparation was performed as described in the previous section. cDNA was diluted 50-fold for use as template. β-actin was selected as the reference gene based on its stability of expression across different life stages of WCR [58]. Primers used for qRT-PCR were designed using Beacon Designer software (Premier Biosoft International, Palo Alto, CA, USA) and are provided in Supplementary Materials Table S1. The 7500 Fast System SDS v.2.0.6 Software was used to determine the slope, correlation coefficients, and efficiencies (Supplementary Materials Table S1). Primer efficiencies were evaluated using 5-fold serial dilutions (1: 1/5: 1/25: 1/125: 1:625) in triplicate. Amplification efficiencies were higher than 96.1% for all the qRT-PCR primer pairs used in this study (Supplemental Materials Table S1). qRT-PCR analysis was performed with three to six biological replicates; each biological replicate had two technical replications. qRT-PCR cycling parameters were set as described in the supplier′s protocol. At the end of each PCR reaction, a melting curve was generated to confirm single peaks and rule out the possibility of primer–dimer and nonspecific product formation. Relative quantifications of the transcripts were calculated using the comparative 2^−ΔΔCT^ method [59] and were normalized to β-actin [9].

### 2.9. Statistical Analysis

Statistical analyses were performed with JMP^®^ Pro 11 [60]. Data were analyzed with a one-way analysis of variance (ANOVA) and the means of the treatments were compared using a Student′s *t*-test with Dunnett′s adjustment (*α*, 0.05).

## 3. Results

### 3.1. brahma and hunchback Concentration Response

**Figure 1:**
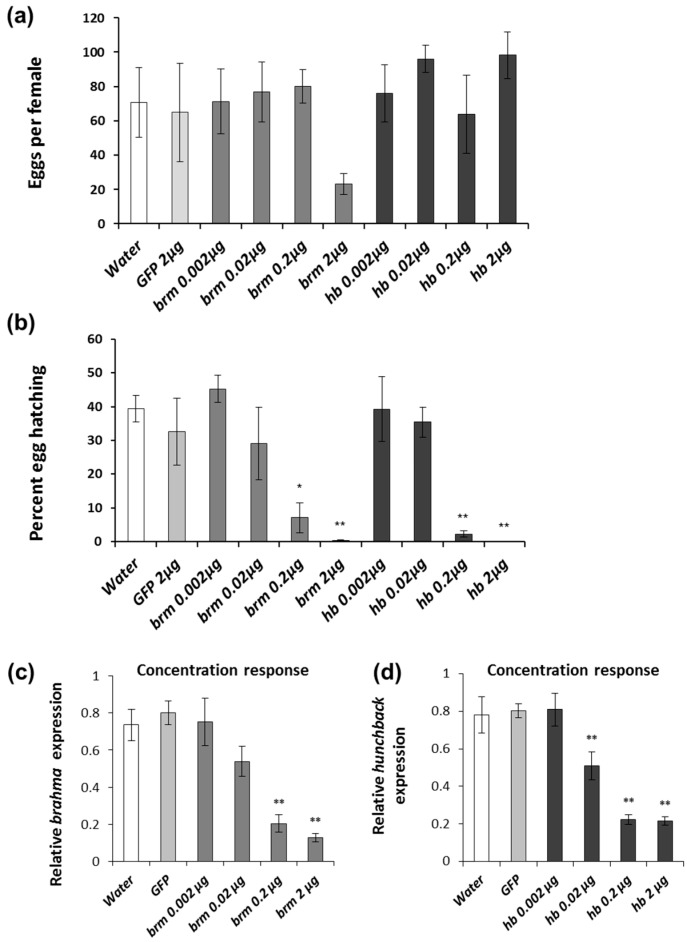
pRNAi concentration response to *brahma* (*brm*) and *hunchback* (*hb*) dsRNA in *Diabrotica virgifera virgifera.* Females were fed with diet treated with 0.002 μg, 0.02 μg, 0.2 μg and 2 μg *brm* or *hb* dsRNA. Diet treated with water or 2 μg *GFP* dsRNA were used as controls. Treatments were applied six times every other day for twelve days. (**a**) Eggs collected from dsRNA-fed females after last feeding exposure; (**b**) Eggs hatched based on numbers oviposited; (**c**,**d**) Relative transcript level for *brahma* (*brm*) and *hunchback* (*hb*) in *D. v. virgifera* females. Comparisons performed with Dunnett′s test, * significance at *p* < 0.05. ** significance at *p* < 0.001.

As previously described, feeding of adult WCR with *brm* or *hb* dsRNA leads to significant reductions in egg hatch rates [16,26]. While the eggs of *brm* dsRNA-fed females showed no signs of embryonic development and appeared as undeveloped or unfertilized eggs; embryos produced by WCR *hb* dsRNA-fed females had missing segments and deformed mouthparts (Supplementary Materials Figure S1) [16]. To determine the lowest concentration necessary to generate a pRNAi response, mated WCR females were exposed a range of concentrations of *brm* and *hb* dsRNA from 0.002 µg to 2 µg of dsRNA per artificial diet pellet six times over twelve days. No significant reduction in the number of eggs per female was observed after females were fed with *brm* dsRNA, although 2 µg of *brm* dsRNA produced a downward trend in oviposition (Figure 1a). Egg production in females exposed to any of the *hb* dsRNA concentrations was unaffected (Figure 1a). A significant reduction in egg hatching was observed with six feedings over a period of 12 days with 0.2 and 2 μg dsRNA for both *brm* and *hb* (Figure 1b). Significant reductions of *brm* transcript levels were detected when females were fed 0.2 and 2 μg of *brm* dsRNA (Figure 1c), while significant reductions of *hb* transcript levels were observed at all three *hb* dsRNA exposure concentrations (Figure 1d). Although a reduction in *hb* expression was observed at the 0.02 μg exposure, there was no reduction in the hatch rate.

### 3.2. Duration of brahma and hunchback dsRNA Exposure

**Figure 2:**
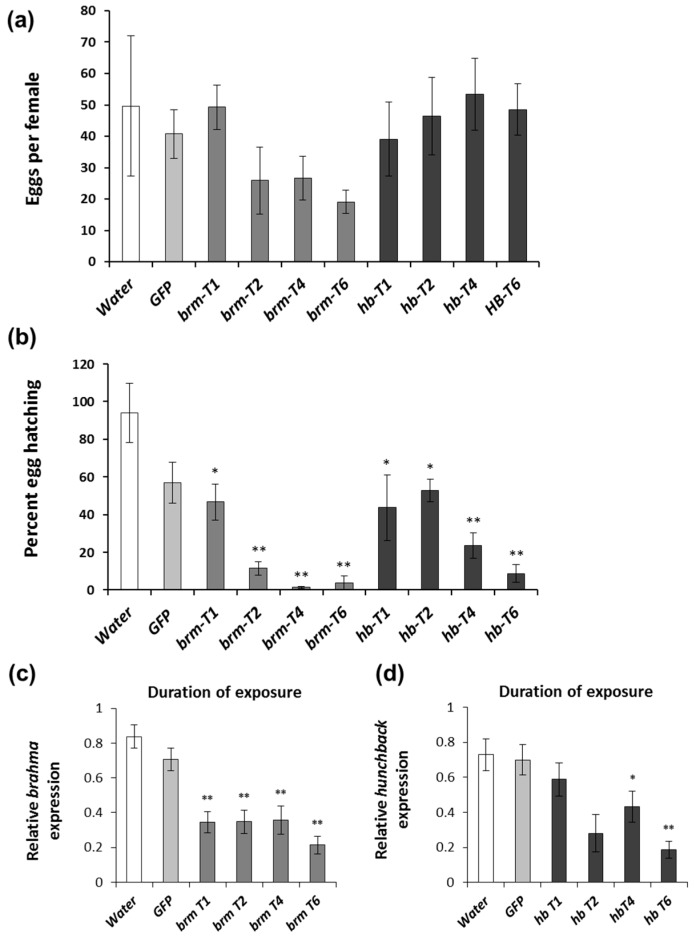
Duration of exposure effects on pRNAi response using *brahma* (*brm*) and *hunchback* (*hb*) dsRNA in *D. v. virgifera.* Females were fed with diet treated with dsRNA; the number following T indicates the number of times that females received dsRNA (2 μg per diet pellet), diet provided every other day for twelve days. Diet treated with water and *GFP* dsRNA provided six times were used as controls. (**a**) Eggs collected from dsRNA-fed females after last feeding exposure; (**b**) Eggs hatched based on numbers oviposited; (**c**,**d**) Relative transcript level for *brahma* (*brm*) and *hunchback* (*hb*) in *D. v. virgifera* females. T indicates the number of times that females received dsRNA (2 μg per diet pellet), diet provided every other day for twelve days. Comparisons performed with Dunnett′s test (control group = water), * significance at *p* < 0.05. ** significance at *p* < 0.001.

Since females feed on a variety of plant material [36,46], it is important to determine the minimal duration of the exposure to dsRNA that generates a pRNAi response. For this purpose, we exposed females to artificial diet treated with 2 μg of dsRNA once, twice, four, or six times, providing freshly treated or untreated diet every other day. Although *brm* dsRNA-fed females showed a downward-trend in oviposition, the number of eggs per female was not significantly different from the water control. Similarly, for the results observed in the concentration response experiment, the egg production of females exposed to *hb* dsRNA was unaffected (Figure 2a). The percentage of larvae hatching was significantly reduced compared to water for all exposures with both genes (Figure 2b). For *brm,* the pRNAi effect was stronger with two to six feedings of 2 μg dsRNA; and for *hb,* at least four feedings were necessary to generate over 50% reduction in egg hatching (Figure 2b). Relative *brm* transcript levels were significantly reduced with all the exposures (Figure 2c), while for *hb* four dsRNA feedings were necessary to observe a significant reduction in *hb* transcript levels (Figure 2d).

### 3.3. Timing of brahma and hunchback dsRNA Exposure with Respect to Mating Status

**Figure 3:**
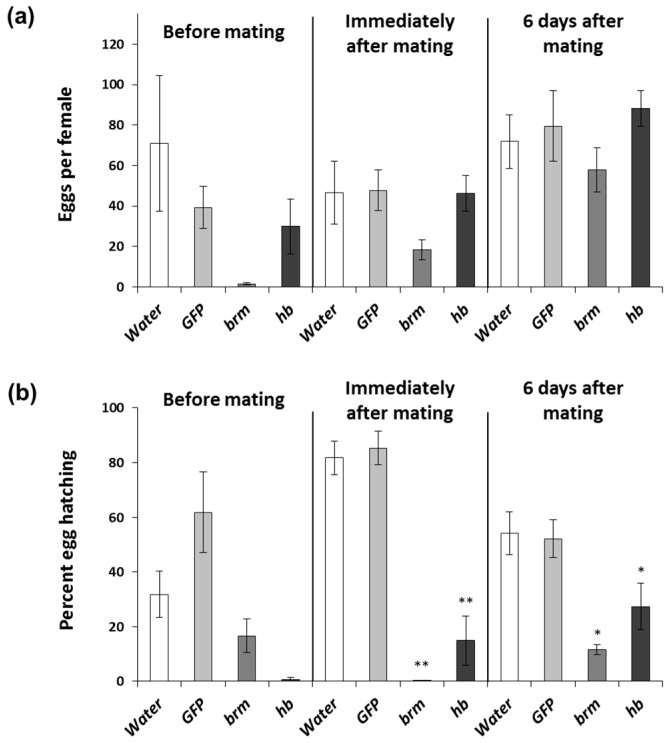
Timing of exposure effects on pRNAi response using *brahma* (*brm*) and *hunchback* (*hb*) dsRNA in *D. v. virgifera.* Females were fed diet with 2 μg dsRNA six times before mating, six times immediately after mating, and six times six days after mating. Diet treated with water and *GFP* dsRNA were used as controls. (**a**) Eggs collected from dsRNA-fed females after last feeding exposure; (**b**) Eggs hatched based on the numbers oviposited. Comparisons performed with Dunnett′s test (control group = water), * significance at *p* < 0.05. ** significance at *p* < 0.001.

**Figure 4:**
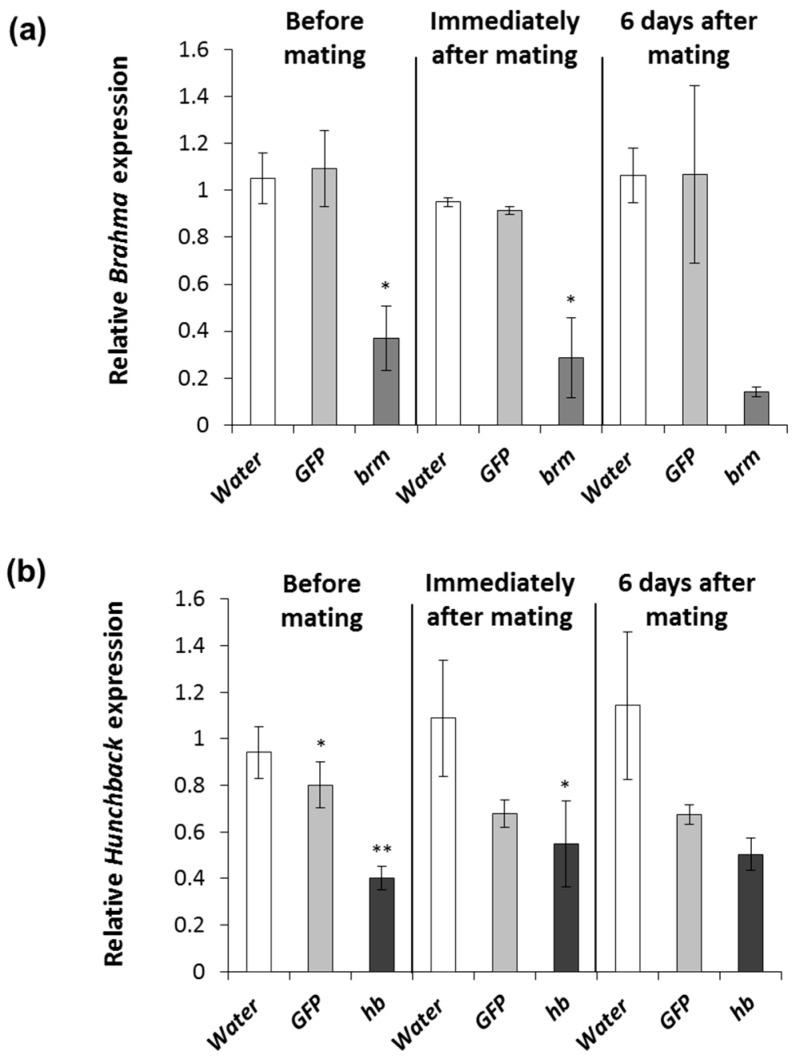
Relative transcript level of *brahma* (*brm*) and *hunchback* (*hb*) in *D. v. virgifera females*: (**a**) relative *brahma* transcript expression for timing of exposure; and (**b**) relative *hunchback* transcript expression from timing of exposure. Comparisons performed with Dunnett′s test (control group = water), * significance at *p* < 0.05. ** significance at *p* < 0.001.

In a field setting, females will be exposed to dsRNA at different times of their reproductive cycle. We evaluated females exposed to 2 μg of *brm* and *hb* dsRNA six times before mating, immediately after mating, and six days after mating to determine the impact of reproductive status on gene expression and phenotypic response. As in the concentration response experiment (Figure 1a), the number of eggs per female was reduced in females exposed to *brm* dsRNA but it was not significantly different from the water control (Figure 3a). In females exposed to *hb* dsRNA egg production was not significantly affected (Figure 3a). The percent eggs hatching was significantly reduced when females were fed *brm* dsRNA immediately after mating and six days after mating; this effect was stronger in females that fed immediately after mating (Figure 3b). Even though the percent of eggs hatching from females fed with *brm* dsRNA before mating was not significantly different from females fed with water treated diet, egg hatching was based on five emerging larvae from a total of 43 eggs, while the total number of eggs for the controls were 2128 and 1406 for the water and *GFP,* respectively (Figure 3b). Low egg hatch was observed in females fed with *hb* dsRNA at any time of their reproductive cycle. The total egg hatch rate was lower when females were fed *hb* dsRNA before mating and immediately after mating (Figure 3b). Relative transcript levels were significantly reduced in females fed *brm* (Figure 4a) and *hb* (Figure 4b) dsRNA before and immediately after mating. Overall, gene knockdown was stronger for *brm* compared to *hb*. To determine if *brm* and *hb* dsRNA treatments caused phenotypic changes in WCR ovary, ovaries of females that were treated with dsRNA before and after mating were dissected; no morphological differences were observed between dsRNA and control treatments (Supplementary Materials Figure S2).

### 3.4. Effects of brahma and hunchback on Males

**Figure 5:**
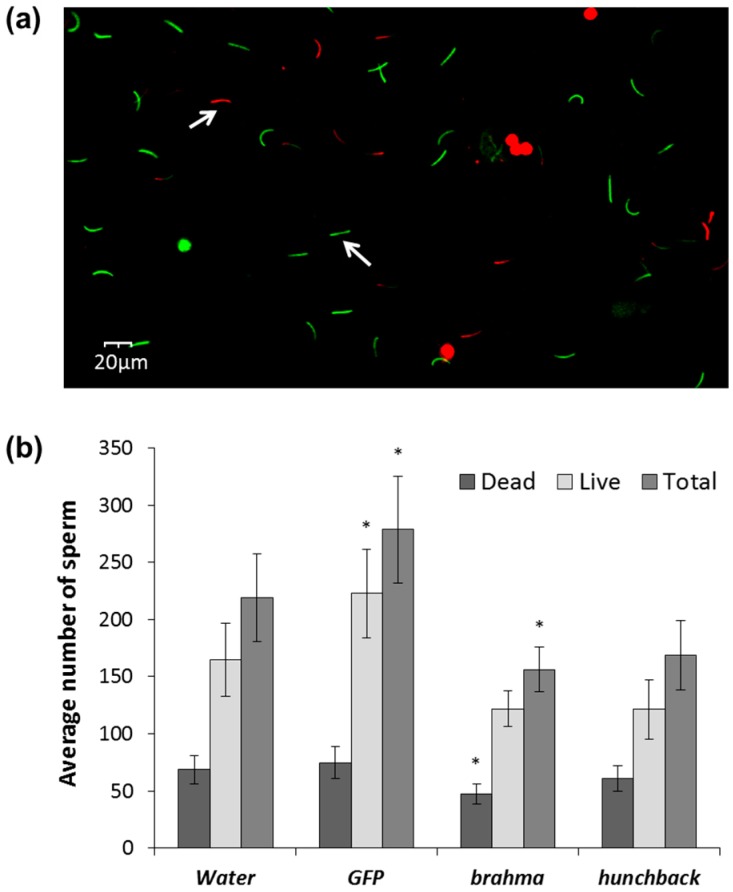
Sperm viability of *D. v. virgifera* males exposed to *brahma* (*brm*) and *hunchback* (*hb*) dsRNA: (**a**) sample composite digital image of live (green) and dead (red) sperm from half of a single field of view (10X); and (**b**) total number of sperm (live + dead) of males exposed four times to *brm* and *hb* dsRNA. Comparisons performed with Dunnett′s test (control group = water), * significance at *p* < 0.05.

**Figure 6:**
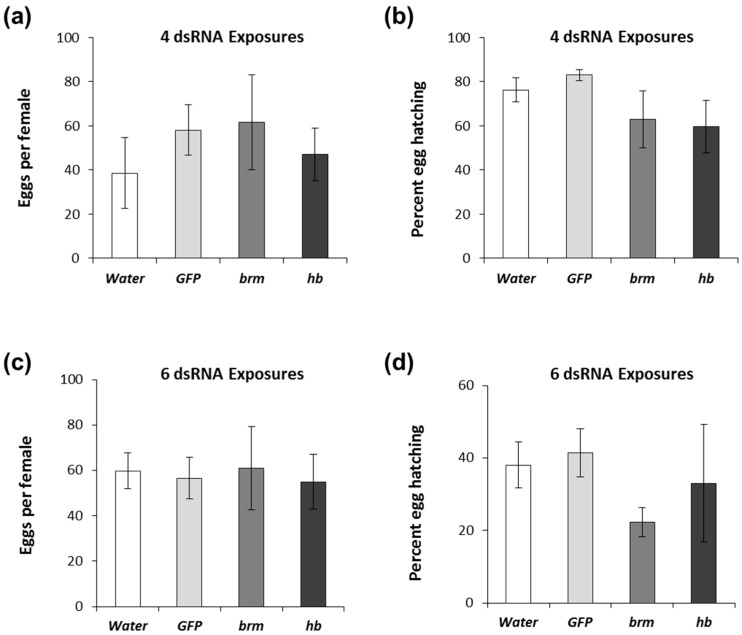
pRNAi response to *brahma* (*brm*) and *hunchback* (*hb*) dsRNA of *D. v. virgifera* males. Males were fed with diet treated with 2 μg dsRNA. Treatment was provided four and six times every other day and mated with females immediately after receiving all dsRNA treatments. (**a**) Eggs collected from females mated with males with four dsRNA exposures; (**b**) Eggs hatched based on numbers oviposited; (**c**) Eggs collected from females mated with males with six dsRNA exposures; (**d**) Eggs hatched based on numbers oviposited. Comparisons performed with Dunnett′s test (control group = water), * significance at *p* < 0.05.

Fertility of males exposed to *brm* and *hb* dsRNA was assessed by testing sperm viability using fluorescent staining techniques [57] and viability of the offspring of males exposed to dsRNA. Sperm cells stained using Live/Dead Viability Kit yielded green fluorescence (500–550 nm) if live and red fluorescence (663–738 nm) if dead (Figure 5a). Results indicated that the overall sperm count was significantly lower in males fed four times with *brm* dsRNA. Whereas males treated with *hb* dsRNA showed a lower live and total number of sperm compared to the controls, although these numbers were not significantly different from water-treated controls (Figure 5b). Even though we observed an overall reduction in the number of sperm, there was no impact on the number of eggs and egg viability from females mated with males exposed to *brm* and *hb* dsRNA after four exposures over eight days (Figure 6a,b) and six exposures over 12 days (Figure 6c,d). A significant reduction of transcript levels was achieved when males were fed *hb* dsRNA but not *brm* when fed dsRNA four times (Supplementary Materials Figure S3a,b); yet significant reduction in transcript abundance was observed when fed dsRNA six times over 12 days for both genes (Supplementary Materials Figure S3c,d). To determine if the differences in gene knockdown between females and males were sex-specific, females and males were fed 2 μg *brm* and *hb* dsRNA four and six times. Gene knockdown for both genes in males was higher or similar to females (Supplementary Materials Figure S3).

## 4. Discussion

The information obtained in this study informs the discussion on dsRNA exposure as it relates to achieving pRNAi responses that may be applied to management of corn rootworm populations. Moreover, this work began to examine the dynamics of the RNAi response in insects that may go beyond pRNAi and crop pests. Adult insects appear to be unaffected by pRNAi targets, allowing estimation of parameters such as the duration of gene knockdown. These parameters are more difficult to measure with genes that affect pigmentation or generate lethality, given that the effects may be confounded by the phenotype itself (i.e., one cannot monitor a recovery of the response once the treated insects are dead; parameters such as recovery in pigmentation may be slower and more difficult to quantify over time). While qRT-PCR may be used to accurately measure gene knockdown [61], it does not take into account protein turnover, which will have profound effects on the outcome or phenotype of the RNAi treatment. Therefore, the use of pRNAi as a model RNAi system may enable a better understanding of the concentration-over-time exposures, onset of the RNAi effect, and interactions of different dsRNA treatments given that changes in both the transcript levels and the phenotype can be quantified. Based on our observations, we postulate that the pRNAi could be used as a model to better understand the RNAi response in insects in general.

The experiments performed with WCR allowed us to quantify the level of exposure to dsRNA that consistently produces a pRNAi response in females. Our results suggest that there is a correlation between the response and the concentration of dsRNA and the exposure time. We observed that the concentration required to generate a reduction in egg hatching for both *brm* and *hb* was at least 0.2 µg per diet pellet with six exposures over twelve days of feeding (Figure 1b). When the dsRNA amount is fixed at 2 µg per diet pellet, the duration of feeding should be of at least four days (two exposures) for *brm* and eight days (four exposures) for *hb* (Figure 2b). In the above experiments, ten WCR females were provided with eleven diet pellets with various amounts of dsRNA every other day. Thus, approximately, 1.1 pellets were provided for each female and the diet pellets were consumed in their entirety in most of the experiments. This setup provides rough estimates of dsRNA consumption per insect and over time (e.g., 1.1 µg of dsRNA per day per female when the dsRNA amount was 2 µg/pellet over two days). Extrapolating from these artificial diet-based observations, females would need to consume approximately 1.1 µg of dsRNA per day over a four-day period (4.4 µg total/female), 0.11 µg per day over a twelve-day period (1.32 µg total/female) or a combination of dose and duration that equals these parameters. Interestingly, we observed that six exposures at 0.2 µg dsRNA (1.32 µg/female) (Figure 2b) were more efficacious than a single exposure of 2 µg of dsRNA (2.2 µg/female) (Figure 2b). Since the single-exposure experiments lasted for the same period of time as the three-exposure experiments, the protein half-life is not the likely explanation for the difference. To determine the benefits of prolonged low-concentration dsRNA exposure vs. acute high-dose dsRNA application more detailed studies need to be performed.

Earlier studies have demonstrated robust and highly sensitive lethal RNAi response in WCR adults [9,62]. In the aforementioned studies, a similar WCR adult feeding approach was used. The LD_50_ for *v-ATPase A* was found to be ~500 ng/diet pellet of dsRNA, applied six times over twelve days [9,62]. For pRNAi, the 2 μg/diet pellet application is four times higher than LC_50_ of a lethal gene. However, is important to consider that *v-ATPase* genes are highly expressed in the WCR midgut [63], while *brahma* and *hunchback* are expressed in the ovaries. The RNAi response in WCR has been found to be systemic [63], hence the movement of dsRNA from the midgut to the ovaries could explain the higher amount of dsRNA needed for pRNAi genes. Additionally, the dose needed to trigger a pRNAi response in this study may reflect the lower sensitivity of the ovary to RNAi or the dose-response of the specific genes used to probe pRNAi in WCR.

It was recently postulated that there could be competition of siRNA and miRNA pathways [64]. Interestingly, in WCR, even at high doses, application of pRNAi or non-lethal dsRNA targets does not produce observable fitness effects [62]. It is also possible that even if the miRNA pathway is affected in response to dsRNA, the miRNA pathway may not be essential during the adult stages of WCR. The observations that *brm* and *hb* dsRNA treatments cause primarily egg hatch defects and no or low-level reduction in oviposition is consistent with no observable changes seen in the morphology of ovaries (Supplementary Materials Figure S2). In an earlier study, we observed *brahma* dsRNA-induced oviposition and ovary development phenotypes in the stink bug, *Euschistus heros* [26], hence the low-level oviposition phenotype in WCR was not surprising. Brahma and other chromatin remodeling ATPases are known to play various roles in oogenesis, early and late embryogenesis [65,66,67,68,69]. The difference in the effects of *brahma* dsRNA on oviposition in WCR and *E. heros* may stem from the differences in the function of these genes between different insect orders. Further, the parental RNAi approach for pest insect control does not necessarily exclude lethality. In the present study, the absence of strong morphological or lethal phenotypes in the adult insect enables a more accurate characterization of the pRNAi response. However, the best plant protection may be achieved by an RNAi trait that confers both lethal and parental effects.

The amount of plant material consumed by WCR is likely to vary depending on the nutritional value of the plant tissue and other biotic and abiotic factors that may affect rates of consumption. Therefore, the best studies to estimate the minimum in-plant dsRNA concentrations for a robust pRNAi response should be performed directly with hpRNA-expressing plant materials. In addition to dose and duration, important factors that need to be considered for successful field exposure include feeding, mating, and dispersal behaviors. Considering that adult rootworms can utilize a variety of plant materials as their food sources [36,37] and can readily move between transgenic, non-transgenic fields [70] as well as native weed species [36], it is likely that adults will not feed exclusively on a single plant. However, the strong fidelity of WCR to maize fields, and the fact that WCR females usually feed on maize tissue after emergence, during mating, or immediately after mating [30] suggest that pRNAi could potentially reduce fecundity in a field setting.

Our results suggest that females were more sensitive to pRNAi before mating and the sensitivity of the response seemed to decrease as the adult females aged. This suggests that for a stronger pRNAi response females should preferably feed on dsRNA before mating and immediately after mating, although a decline in egg hatching was still observed in females that fed after mating. Based on these results, pRNAi would be most successful if females feed on dsRNA before or immediately after mating. This will align well to the behaviors observed in the field, given that females feed on maize tissues immediately after mating to stimulate egg development [4,71]. In addition, after mating, WCR females tend to remain in their natal maize field for several days before dispersal [72]; this would be in the range of the four-day exposure that we tested in the lab. Females emerging from adjacent maize fields that do not express pRNAi and migrate to a field expressing a pRNAi trait will be exposed later in their reproductive development. Since we observed pRNAi phenotypes in females that were exposed to pRNAi six days after mating, even shorter exposures or exposure several days after mating may produce pRNAi effects. Given that only 5%–10% of eggs successfully establish in the field [73], greenhouse or field-based testing will be suited best to answer these more complex scenarios. In practice, the concept of pRNAi would best be implemented in maize plants in combination with Bt toxins and/or RNAi lethal genes so any emerging larvae will be potentially killed by maize expressing a Bt toxin and/or lethal RNAi. In the above pyramid, pRNAi would serve the function of an added control measure to extend durability [13,26].

Unlike the robust fecundity phenotypes observed in females, no egg viability defects were detected after dsRNA treatments of males. A decrease in the total number of sperm was observed after exposure of males to *brm* and *hb* dsRNA, however this decrease may not be enough to result in measurable changes in male fertility.

The experiments performed in this study provided a means to quantify the level of exposure to dsRNA that consistently produces a pRNAi response in exposed WCR females; this will assist in establishing a baseline for the potential efficacy of transgenic maize plants expressing hpRNA for pRNAi target sequences. The next step for the validation of this technology will be testing the efficacy of maize plants expressing long hairpin RNA for *brm* and *hb* and to correlate the effects of the successful pRNAi exposure parameters to behaviors of WCR adults. Furthermore, because females have been reported to oviposit for up to 60 days during their lifespan [42,74], it will be important to validate the onset and the longevity of the pRNAi response in WCR females. Further research evaluating the effects of *brm* and *hb* on larval survival, development, and the ability of larvae exposed to parental RNAi to produce offspring will provide a better understanding of the pRNAi response and its potential use for corn rootworm management.

The benefits of pRNAi for crop protection reach beyond WCR. In a recent publication, we demonstrated that dsRNA can generate a strong pRNAi response in the Neotropical brown stink bug *E. heros*, by injection [26]. For insects like stink bugs that have multiple generations per year, the use of a pRNAi strategy will have most benefit since it will control insects within the same season. However, there is no oral response in stink bugs. Once the barriers to oral delivery to lepidopteran and hemipteran insects are overcome, multiple pest management areas, particularly for multivoltine pests, may benefit from pRNAi.

## 5. Conclusions

In conclusion, this study has probed the concentration, duration, and timing of the exposure needed for a successful pRNAi response in WCR. Described herein, diet-based RNAi studies have an advantage in that the amount of dsRNA is tightly controlled, enabling basic research pertaining to the concentration and the exposure parameters of pRNAi and RNAi in general. These experiments provide a framework for plant-based testing of pRNAi, allowing for a more accurate assessment of the potential of pRNAi, when applied at the field level. A path forward for pRNAi as a pest management tool will build on this work to ascertain efficacy in transgenic plants and the longevity of the pRNAi effect. Furthermore, *brm*, *hb*, and potentially other pRNAi also provide a platform to better understand RNAi in insects.

## Supplementary Materials

The following are available online at www.mdpi.com/2073/4425/8/1/7/s1, Figure S1: Parental *hunchback* dsRNA phenotypes in *D. v. virgifera*, Figure S2: Ovaries of *hunchback* and *brahma* dsRNA-fed *D. v. virgifera*, Figure S3: Comparison of relative transcript level for *brahma* (*brm*) and *hunchback* (*hb*) between *D. v. virgifera* females and males, Table S1: Primer pairs used to amplify DNA templates for *D. v. virgifera* dsRNA synthesis and qRT-PCR.
