# Supplementary material for: Parameters for Successful Parental RNAi as An Insect Pest Management Tool in Western Corn Rootworm, Diabrotica virgifera virgifera
Source: Genes (Basel). 2016 Dec 24;8(1):7. doi: 10.3390/genes8010007 (PMC5295002; doi:10.3390/genes8010007)
Supplement: Supplementary file 1 [file genes-08-00007-s001.pdf]

Supplementary Materials: Parameters for Successful Parental RNAi as An Insect Pest Management Tool in Western Corn Rootworm, Diabrotica virgifera virgifera

Ana M. Vélez, Elane Fishilevich, Natalie Matz, Nicholas P. Storer, Kenneth E. Narva, and Blair D. Siegfried

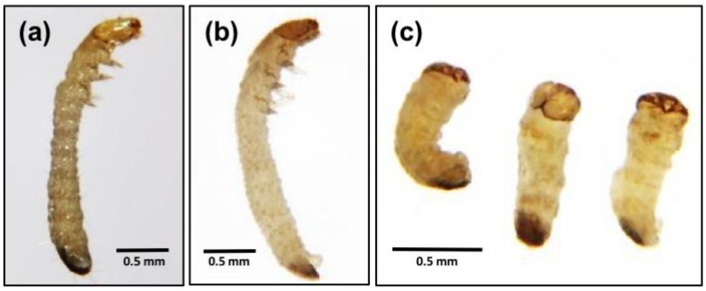

Figure S1. Parental *hunchback* dsRNA phenotypes in *D. v. virgifera*. *D. v. virgifera* females were fed with diet treated six times with 2 µg *hunchback* dsRNA. (**a**) Developed larva from a female fed with untreated diet; (**b**) Developed larva from an *GFP* dsRNA-fed female. (**c**) Larvae dissected from eggs of a *hb* dsRNA-fed female. The larvae exhibit a "hunchback" phenotype that includes fewer segments, missing limbs and deformed mouth parts.

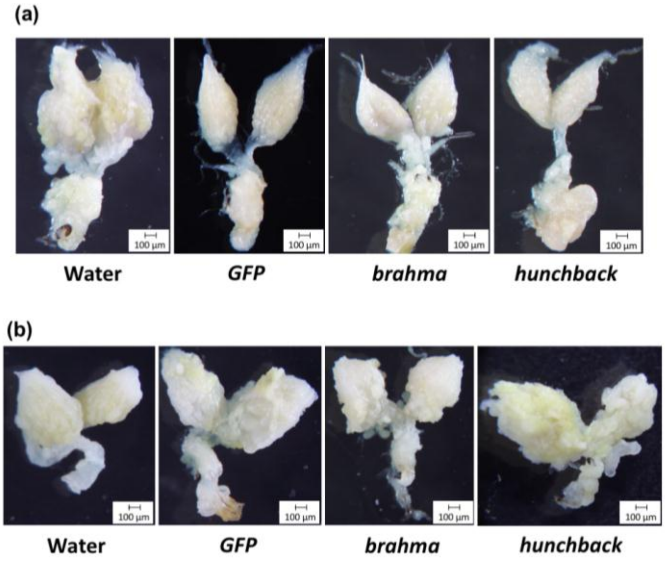

Figure S2. Ovaries of *hunchback* and *brahma* dsRNA-fed *D. v. virgifera*. Ovaries of females fed diet treated with water, 2 µg of GFP, *brahma* or *hunchback* dsRNA six times. Diet provided every other day for 12 days. Dissections performed one day after receiving the last dsRNA treatment. (**a**) Ovaries of females fed dsRNA before mating; (**b**) Ovaries of females fed dsRNA after four days of mating.

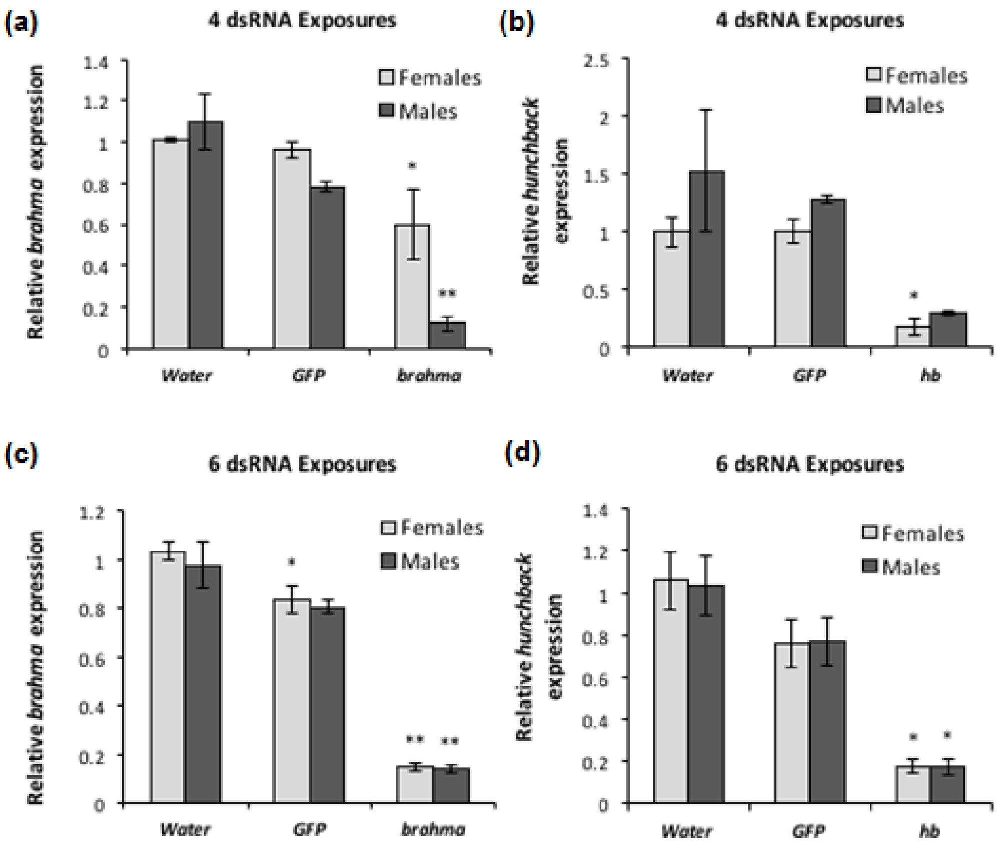

Figure S3. Comparison of relative transcript level for *brahma* (*brm*) and *hunchback* (*hb*) between *D. v. virgifera* females and males. (**a**) Relative *brahma* transcript expression for females and males exposed four times to *brm* dsRNA; (**b**) Relative *hunchback* transcript expression for females and males exposed four times to *hb* dsRNA; (**c**) Relative *brahma* transcript expression for females and males exposed six times to *brm* dsRNA; (**d**) Relative *hunchback* transcript expression for females and males exposed six times to *hb* dsRNA. Three biological replications per treatment and sex. Comparisons within each sex were performed with Dunnett's test (control group = water), * significance at $p < 0.05$. ** significance at $p < 0.001$. Comparisons between sexes within each treatment were performed with Student's *t*-test, * significant differences at $p < 0.05$.

Table S1. Primer pairs used to amplify DNA templates for *D. v. virgifera* dsRNA synthesis and qRT-PCR. Product size for dsRNA excludes T7 sequence [1].

Gene Name	Primer Sequences for dsRNA Synthesis				Product Length (bp)
brahma	Forward: TAATACGACTCACTATAGGGAACCTTCTTCATCTTCTG<br>Reverse: TAATACGACTCACTATAGGGCTCTCCTAATACAGTTCAA				352
hunchback	Forward: TAATACGACTCACTATAGGGAAGTGTAAGCAATGTGATT<br>Reverse: TAATACGACTCACTATAGGGTTATGGTACAAGGAGAGGA				405
GFP	Forward: TAATACGACTCACTATAGGGGGTGATGCTACATACGGAAAG<br>Reverse: TAATACGACTCACTATAGGGTTGTTTGTCTGCCGTGAT				370
**Gene Name**	**Primer Sequence for qRT-PCR**	**Product Length (bp)**	**Slope**	**R^2**	**Primer Efficiency (%)**
brahma	Forward: TCGCTTGATTCTGCTTGTGGA<br>Reverse: AGAACGAAGCGACAGGGTCT	166	−3.266	0.996	100.41
hunchback	Forward: TGCCCCAAGTGTCCTTTTGT<br>Reverse: CAGTCAGAACAGCGGTATTGGT	179	−3.348	0.997	98.94
β-actin	Forward: TCCAGGCTGTACTCTCCTTG<br>Reverse: CAAGTCCAAACGAAGGATTG	134	−3.419	0.999	96.1

Underlined sequence corresponds to T7 promoter.

Reference

1. Khajuria, C.; Vélez, A.M.; Rangasamy, M.; Wang, H.; Fishilevich, E.; Frey, M.L.F.; Carneiro, N.; Premchand, G.; Narva, K.E.; Siegfried, B.D. Parental RNA interference of genes involved in embryonic development of the western corn rootworm, *Diabrotica virgifera virgifera* LeConte. *Insect Biochem. Mol. Biol.* **2015**, *63*, 54–62.
